# Community-level risk factors for notifiable gastrointestinal illness in the Northwest Territories, Canada, 1991-2008

**DOI:** 10.1186/1471-2458-13-63

**Published:** 2013-01-22

**Authors:** Aliya Pardhan-Ali, Jeff Wilson, Victoria L Edge, Chris Furgal, Richard Reid-Smith, Maria Santos, Scott A McEwen

**Affiliations:** 1 Department of Population Medicine, University of Guelph, Guelph, ON, Canada; 2 Novometrix Research Inc, Moffat, ON, Canada; 3 Department of Indigenous Environmental Studies, Trent University, Peterborough, ON, Canada; 4 Department of Health and Social Services, Government of the Northwest Territories, Yellowknife, NT, Canada

**Keywords:** Ecology, Risk factors, Regression analysis, Minority health, Gastrointestinal diseases

## Abstract

**Background:**

Enteric pathogens are an important cause of illness, however, little is known about their community-level risk factors (e.g., socioeconomic, cultural and physical environmental conditions) in the Northwest Territories (NWT) of Canada. The objective of this study was to undertake ecological (group-level) analyses by combining two existing data sources to examine potential community-level risk factors for campylobacteriosis, giardiasis and salmonellosis, which are three notifiable (mandatory reporting to public health authorities at the time of diagnosis) enteric infections.

**Methods:**

The rate of campylobacteriosis was modeled using a Poisson distribution while rates of giardiasis and salmonellosis were modeled using a Negative Binomial distribution. Rate ratios (the ratio of the incidence of disease in the exposed group to the incidence of disease in the non-exposed group) were estimated for infections by the three major pathogens with potential community-level risk factors.

**Results:**

Significant (p≤0.05) associations varied by etiology. There was increased risk of infection with *Salmonella* for communities with higher proportions of ‘households in core need’ (unsuitable, inadequate, and/or unaffordable housing) up to 42% after which the rate started to decrease with increasing core need. The risk of giardiasis was significantly higher both with increased ‘internal mobility’ (population moving between communities), and also where the community’s primary health facility was a health center rather than a full-service hospital. Communities with higher health expenditures had a significantly decreased risk of giardiasis. Results of modeling that focused on each of *Giardia* and *Salmonella* infections separately supported and expanded upon previous research outcomes that suggested health disparities are often associated with socioeconomic status, geographical and social mobility, as well as access to health care (e.g. facilities, services and professionals). In the campylobacteriosis model, a negative association was found between food prices in communities and risk of infection. There was also a significant interaction between trapping and consumption of traditional foods in communities. Higher rates of community participation in both activities appeared to have a protective effect against campylobacteriosis.

**Conclusions:**

These results raise very interesting questions about the role that traditional activities might play in infectious enteric disease incidence in the NWT, but should be interpreted with caution, recognizing database limitations in collection of case data and risk factor information (e.g. missing data). Given the cultural, socioeconomic, and nutritional benefits associated with traditional food practices, targeted community-based collaborative research is necessary to more fully investigate the statistical correlations identified in this exploratory research. This study demonstrates the value of examining the role of social determinants in the transmission and risk of infectious diseases.

## Background

The number of published studies examining the role of contextual community-level factors (e.g., socioeconomic, cultural and physical environmental conditions) on notifiable gastrointestinal illness (NGI) has recently grown, yet research exploring how such factors influence disease incidence in the Northwest Territories (NWT) is limited [1-6]. Though consistent and significant associations have been found with income, education, household size and medical care-seeking behaviors [1,2], there has been little examination of cultural and subsistence practices including harvesting, processing, sharing and consuming animals, fish and plants (i.e., country or traditional foods) as determinants of the incidence of NGI [3-6].

The NWT is a federal territory in northwestern Canada [7]. There are 33 officially recognized communities; of these, most have fewer than 1,000 residents and more than one-third are remote (more than 350 kilometers away from an urban center) and/or isolated (only accessible by air or during winter months, by ice roads) [8].

As of the 2006 Census, the population was 41,464, an increase of 11% from 2001, with a majority Aboriginal population (50.3%) [7]. About 61% (12,640) of all Aboriginal residents in the NWT are First Nations while 20% (4,200) are Inuit and 17% (3,600) are Métis. Yellowknife has the largest Aboriginal (First Nations, Métis and Inuit) population, 4,105 (22.2%). Behchoko has the largest First Nations population, 1,730 (91.5%), and Inuvik has the largest Inuit population, 1,335 (38.9%) [8].

Data on patients with NGI and other notifiable diseases in the NWT are collected in the NWT Communicable Disease Registry (NWT CDR); however, the registry data alone contain little or no patient-level epidemiological data or community-level information. A lack of socioeconomic, behavioral and environmental information at the patient level restricts the evaluation of these variables as potential risk factors for NGI. Linking disease surveillance data with the NWT Community Survey however, provides an opportunity to investigate potential risk factor data at the community level. Using data from both the NWT CDR and the NWT Community Survey, an ecological analysis was conducted to identify various risk factors for the three most commonly reported etiologic agents of NGI in the NWT: *Campylobacter spp., Giardia spp.,* and *Salmonella spp*. A better understanding of how community-level characteristics affect NGI can help inform the development of community-based programs to prevent and control infection.

## Methods

### Study design

Data on 708 patients with laboratory-confirmed NGI were obtained from the NWT CDR. This study focused on those case-patients with illnesses attributed to the three most frequently reported pathogens. Campylobacteriosis, salmonellosis and giardiasis accounted for 82% of the 708 NGI reported from 33 communities during January 1, 1991 through December 31, 2008: 175 (25%), 202 (28%) and 205 (29%) respectively. Ethics approval was obtained from the University of Guelph Research Ethics Board, the Government of the Northwest Territories and the Aurora Research Institute.

### Data sources

The three dependent variables used for risk factor analyses were community-level incidence rates of laboratory-confirmed campylobacteriosis, giardiasis and salmonellosis. Eighteen independent variables from the NWT Community Survey [8] were considered for analysis based on availability, biological plausibility and findings from other research [1,2,6]. These variables are listed and defined in Table 1.

**Table 1:** Independent variables from the NWT Community Survey included in the risk factor analysis of NGI

**Variable name**	**Definition*(8)***
**Households in core need**	Percentage of households that fall below at least one of the adequacy, affordability or suitability standards and that have a total household income below the Community Core Need Income Threshold. Suitability is defined as having the appropriate number of bedrooms for the characteristics and number of occupants as determined by the National Occupancy Standard requirements. Adequate housing must have running water, an indoor toilet, bathing and washing facilities and must not require major repairs. Affordable housing costs less than 30% of household income where shelter costs include utilities, water, heat, insurance, property taxes and lease costs and rent or mortgage payments. The Core Need Income Threshold is an income limit for each community that represents the amount of income a household must have to be able to afford the cost of owning and operating a home or renting in the private market without government assistance.
**No high school education**	Percentage of population 15 years of age or older that do not have a high-school diploma.
**Unemployment rate**	Total number of unemployed persons 15 years of age or older divided by the total number of persons 15 years of age and older participating in the labor force.
**Median income**	Median personal income for persons aged 15 and over, from all sources.
**Single parent families**	Percentage of single-parent families among all married, common law or single parent families living in private households with at least one never-married son or daughter living in the same household.
**Primary health facility**	**Nursing station**: primary care by community health nurses; **health center**: wide range of programs and services for outpatient care; **hospital**: inpatient, outpatient and emergency services*.*
**Health expenditure per capita**	Public expenditure divided by the population. Health expenditure includes the provision of health services (preventive and curative), family planning activities, nutrition activities, and emergency aid designated for health, but excludes the provision of water and sanitation.
**Physician billing per capita**	Collection of fees for public medical services per person per year.
**Drinking water source**	**Ground:** any subsurface water that occurs beneath the water table in soil and other geologic forms; **surface**: water occurring in lakes, rivers, streams or other fresh water sources.
**Water treatment type**	**Small system**: filtration with UV and liquid chlorine disinfection and storage; **class 1**: tempering, gaseous chlorine disinfection and liquid fluoridation; **class 2**: coagulation, flocculation, inclined tube settling and multi-media filtration with liquid chlorine disinfection and storage.
**Waste disposal system**	**Trucked:** liquid sewage pick-up from holding tanks or plastic bags from residences; **piped:** interior plumbing and fixtures for holding and collecting waste.
**Traditional foods**	Percentage of households reporting that most or all (75% or more) of the meat or fish consumed is harvested.
**Hunting/fishing**	Percent of people 15 years of age or older that hunted or fished during the year.
**Food price index**	Consists of the average of 6 commodity group prices indices (meat, dairy, cereals, oils and fats, sugar) weighted with the average export shares of each of the groups.
**Trapping**	Percent of people 15 years of age or older that trapped during the year
**Population density**	Total population of community divided by its area in km^2^.
**Internal mobility**	Percentage of population moving between communities within the last one year.
**Rural**	An area with a population of less than 1,000 and a density of less than 400 persons per km^2^.

### NWT communicable disease registry

Campylobacteriosis, giardiasis and salmonellosis are included in the NWT Communicable Disease Schedule [9]. The NWT Communicable Disease Manual provides guidelines to assist public health practitioners with decision-making about specific situations and to support the consistency of territorial public health practice [9], therefore the general procedures for notification remained consistent over the study period. Upon symptomatic presentation of NGI (as described in the Manual), health practitioners sent the patient’s clinical specimen to the laboratory for confirmation and serotyping. They also collected the patient’s demographic (age, gender and community), food and water history data for submission to the Population Health Division of the Government of the Northwest Territories Department of Health and Social Services. Health practitioners and laboratories were required to report patients with confirmed campylobacteriosis, giardiasis and salmonellosis to the Population Health Division within 24 hours. Information about patients with laboratory-confirmed NGI was entered into the NWT CDR [9].

### NWT community survey

The NWT Community Survey is carried out by the NWT Bureau of Statistics every fourth and ninth year of a decade. The goal of the Community Survey is to increase the frequency of data collected at the community level and to ask questions relevant to the NWT, particularly about Aboriginal populations, which are not addressed in the Census of Canada. Community-level data were available for 1989, 1994, 1999, 2004, and 2009 and therefore, all of the surveys were used for this study. For non-survey years, the population data closest to the year of diagnosis were used for the study. Each survey included on average, a random sample of 4,000 households and 13,000 persons (one-third of the population) across the territory. Community was the smallest geographic unit for which data were available for public use. A community was defined as a stable statistical subdivision that is relatively homogeneous with respect to population characteristics, economic status and living conditions [8]. Information was available for all 33 communities in the NWT.

### Statistical analyses

The analyses were conducted using the Statistical Analysis System (SAS) for personal computers, version 9.2 (SAS Institute Inc., Cary, NC, USA). Poisson regression was used to model associations between community-level predictors and the outcomes (incidence rates of campylobacteriosis, giardiasis and salmonellosis). Random effects are summarized on the basis of their estimated variances/covariances and, in this case, took the form of random intercepts for each participating community. Separate models were developed for the three outcomes to examine whether different diseases had different risk factors. Predictors were evaluated on an individual basis (through unconditional analyses) for association with dependent variables and were considered for inclusion in the multivariable model if the p-value was ≤0.20. Many of the variables with p≤0.20 were highly correlated with each other (0.8≤ρ≤0.9) and, in these instances the variable with the fewest missing observations was retained for further analysis. Quadratic terms of continuous variables were used to test the assumption of linearity and included if the effect was significant by the *t*-test at p≤0.05 (indicating nonlinearity) to correct for a nonlinear relationship. Biologically plausible two-way interaction terms were tested with the outcome and retained if significant by the *t*-test at p≤0.05. Manual backward stepwise analysis was used to build a final mixed model. Independent variables (risk factors) were retained in the final model if significant by the *t*-test at p≤0.05, or in the case of confounders, if inclusion of the variable (regardless of statistical significance) resulted in a change in the measure of effect by ≥20%. Model fit was evaluated by comparing the ratio of the generalized chi-square statistic to the model degrees of freedom. If the statistic suggested that there was overdispersion (variance was greater than the mean), a Negative Binomial model was fitted to the study data. If the over-dispersion parameter was zero, this indicated that the Negative Binomial distribution was equivalent to a Poisson distribution. If the over dispersion parameter was significantly different from zero, this reinforced that the Negative Binomial distribution was more appropriate by allowing the variance to be greater than the mean. The rate of campylobacteriosis was modeled using a Poisson distribution whereas the rates of giardiasis and salmonellosis were modeled using a Negative Binomial distribution. Measures of association were expressed as rate ratios (the ratio of the incidence of disease in the exposed group to the incidence of disease in the non-exposed group), and the z-statistic was used to calculate 95% confidence intervals.

## Results

### Campylobacteriosis

Of the 18 variables evaluated in the unconditional analysis, seven were significant when the p-value was ≤0.20 on initial univariable screening: rural, hunting/fishing, traditional foods, trapping, food price index, no high school education, and physician billing per capita.

**Figure 1:**
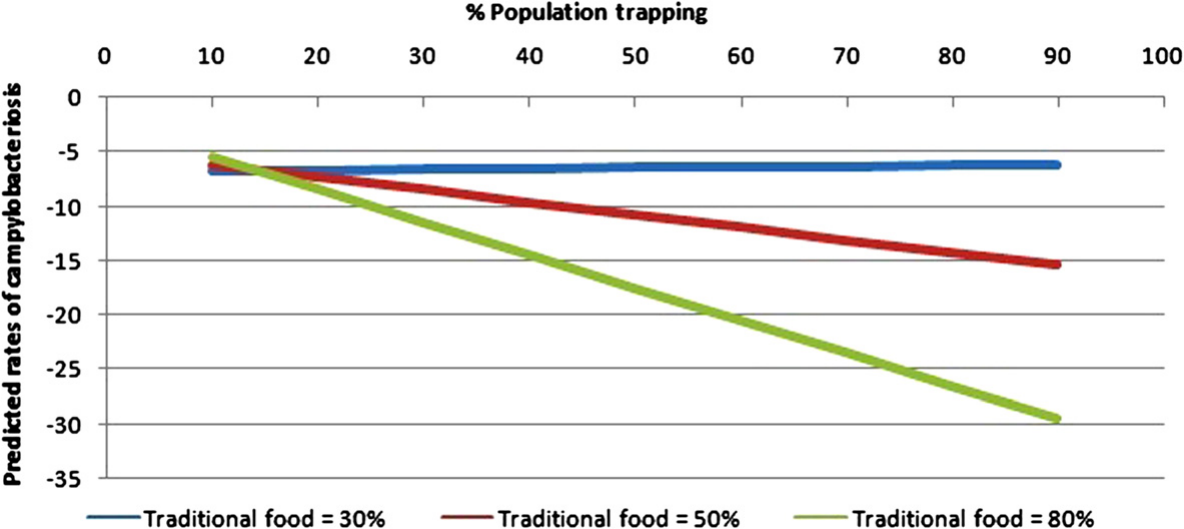
**Interactive effects of traditional food consumption and trapping on rates of campylobacteriosis in the NWT.** Consumption of traditional foods was classified as low, medium and high, representing 30%, 50% and 80% of all food consumed, respectively. When the percentage of community participation in trapping was low, the risk of campylobacteriosis varied little with increasing percentages of traditional food consumption; however, when the percentage of community participation in trapping was medium to high, the protective effect against campylobacteriosis increased with greater consumption of traditional foods.

In the final model, the significant variables (p≤0.05) were traditional foods, food price index, and an interaction term between traditional foods and trapping [traditional foods x trapping]. The model parameter estimates and associated statistics are shown in Table 2. The rate of infection decreased by 3.3% for every 1 unit increase in food price index (protective effect). Due to the presence of a significant interaction term [traditional foods x trapping], the main effects of traditional foods and trapping could not be interpreted on their own. To interpret the interactive effects, Figure 1 plots the percentage of trappers against the risk of campylobacteriosis with consumption of traditional foods classified as low, medium and high, representing 30%, 50% and 80% of all food consumed, respectively. The graph showed that when the trapping percentage was low, the risk of infection varied little with increasing percentages of traditional food consumption; however, when the trapping percentage was medium or high, the protective effect increased with greater consumption of traditional foods.

**Table 2:** Final regression models of significant (p≤0.05) community-level risk factors for campylobacteriosis (Poisson), giardiasis (Negative Binomial) and salmonellosis (Negative Binomial) in the NWT

**Risk factor by disease**		**Estimate**	**Rate ratio**	**Standard error**	**95% Confidence interval**		**Pr< |t|**
**Campylobacteriosis (n=175)**							
Intercept		-6.050	0.002	1.130	<0.0001	0.022	<0.0001
Trapping		0.238	1.269	0.125	0.993	1.622	0.057
Traditional food		0.084	1.087	0.033	1.020	1.159	0.010
Food price index		−0.033	0.967	0.010	0.948	0.989	0.001
Trapping*traditional food		−0.006	0.994	0.003	0.989	0.999	0.022
**Giardiasis (n=205)**							
Intercept		−6.780	0.001	0.530	<0.0001	0.003	<.0001
Health expenditure per capita		−0.052	0.949	0.015	0.922	0.978	0.001
Primary health facility	Center	0.616	1.852	0.250	1.136	3.021	0.014
	Station	−1.155	0.315	1.049	0.040	2.462	0.271
	Hospital	0.000	1.000	.	.	.	.
Internal mobility		0.029	1.030	0.014	1.008	1.059	0.044
**Salmonellosis (n=202)**							
Intercept		−8.054	<.0001	0.486	<.0001	0.001	<.0001
Core need		0.086	1.090	0.032	1.024	1.159	0.007
Core need*core need		−0.001	0.999	.0001	0.998	0.999	0.026

### Giardiasis

Of the 18 variables evaluated in the unconditional analysis, four were significant when the p-value was ≤0.20 on initial screening: internal mobility (population moving between communities), unemployment, health expenditure per capita and primary health facility.

In the final model, the significant predictors (p≤0.05) were internal mobility (population moving between communities), primary health facility and health expenditures per capita. The model parameter estimates and associated statistics are shown in Table 2. The rate of infection increased by 3% for every 1% increase in internal mobility (population moving between communities) in the past year. The rate of giardiasis decreased by 5.1% for every $100 increase in health expenditure per capita (protective effect). The rate of disease was 85.2% higher in communities where the primary health facility was a health center rather than a full-service hospital.

### Salmonellosis

Of the 18 variables evaluated in the unconditional analysis, eight were significant when the p-value was ≤0.20 on initial screening: rural, internal mobility (population moving between communities), households in core need (unsuitable, inadequate and/or unaffordable housing), single parent families, no high school education, median income, unemployment, and hunting/fishing.

**Figure 2:**
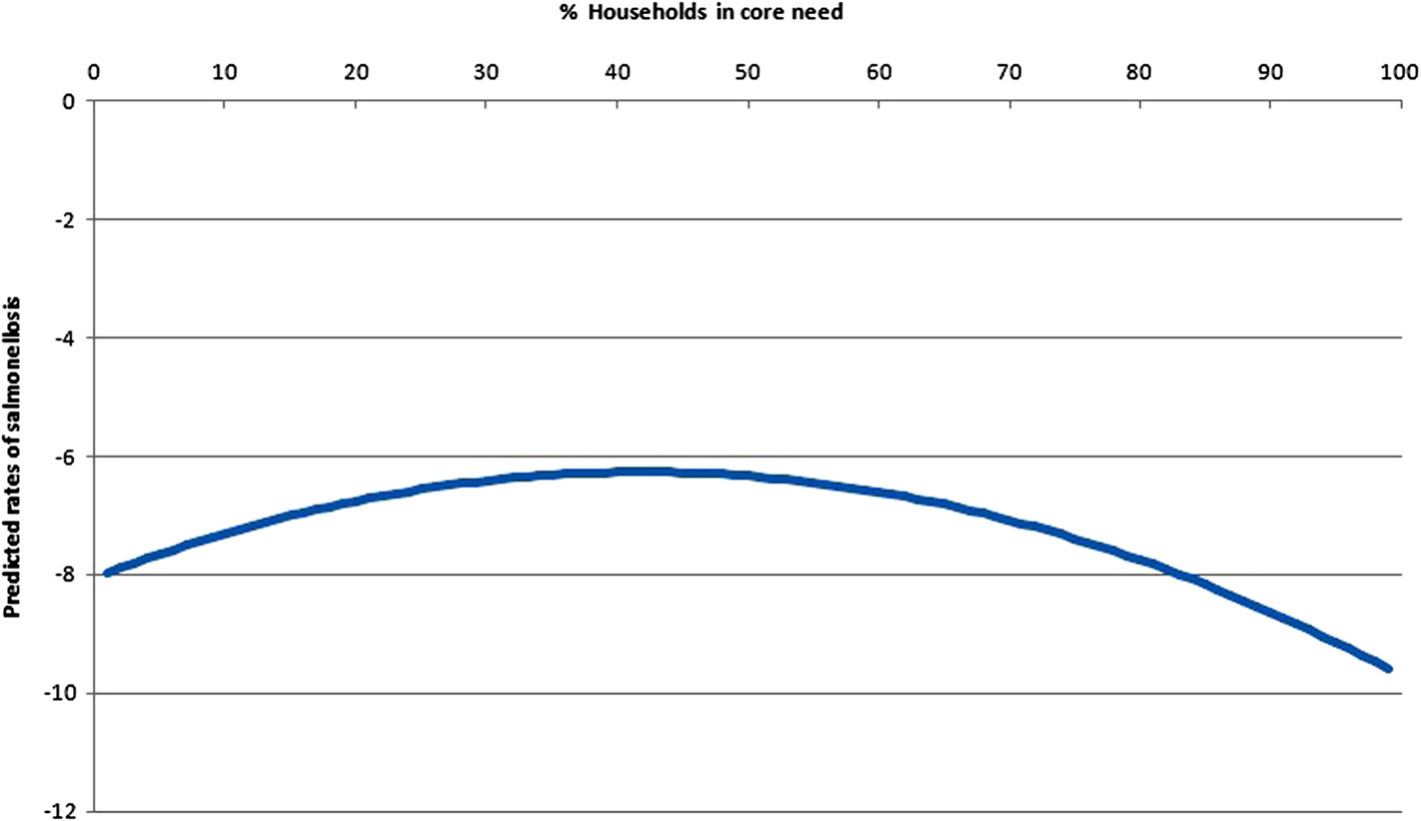
**Quadratic (nonlinear) effect of households in core need on rates of salmonellosis in the NWT.** The nonlinear relationship indicated that the effect of core need households on risk of salmonellosis was unlikely to remain the same as the percentage increased in communities. The rate of infection increased with each 1% increase in core need households in communities up to 42.2% after which the rate started to decrease with increasing core need.

Households in core need and its quadratic term [core need x core need] were the only significant (p≤0.05) predictors in the final model. The model parameter estimates and associated statistics are shown in Table 2. Since there was a quadratic term in the model, the estimate for core need had an interpretation that was different from that in ordinary linear models (the effect of a unit change in its associated variable, holding all other variables constant). Figure 2 plots the percentage of core need households against the risk of salmonellosis. The nonlinear relationship indicated that the effect of core need households on risk of salmonellosis was unlikely to remain the same as the percentage increased in communities. The rate of infection increased with each 1% increase in core need households in communities up to 42.2% after which the rate started to decrease with increasing core need.

## Discussion

This study examined the relationships between potential community-level risk factors and rates of the three most frequently reported enteric infections in the NWT from 1991 through 2008. Significant (p≤0.05) associations seemed to vary by etiology of NGI: campylobacteriosis was primarily associated with cultural and subsistence practices (traditional food consumption, trapping and food price index); giardiasis was mainly associated with health system factors (primary health facility, health expenditure per capita and internal mobility); and salmonellosis was only associated with households in core need (surrogate for socioeconomic status). Campylobacteriosis, giardiasis and salmonellosis are infections that are mainly acquired from the environment, for example, from food, water, and contact with animals or birds; therefore, the significant statistical correlations in the models could be considered as representing indicators of opportunities for exposure rather than direct cause-effect relationships.

Giardiasis was the most commonly reported infection by public health authorities in the NWT. Giardiasis is often contracted through the ingestion of infective cysts found in contaminated water, food, or infected persons by the fecal-oral route. The cysts can be present in contaminated wells and water systems, particularly those sourced from surface water such as fresh water lakes, rivers, and streams [10]. The consumption of raw (untreated) surface water during cultural, economic or recreational activities could be a possible explanation for higher rates of giardiasis with increased internal mobility (population moving between communities). Person-to-person transmission also accounts for many *Giardia* infections especially in child care settings, in public institutions, or among those residing in areas with poor sanitation and hygiene [11]. Although the role of animals in the transmission of human giardiasis is unclear, about 40% of NWT residents spend time on the land carrying out subsistence activities which can result in increased exposure to pathogenic agents in the physical environment and also in wild animals [8,12]. Cysts of *Giardia spp.* have been found in water, sewage and fecal samples of marine mammals harvested for food in the Arctic [13]. In particular, seals have been suggested as one of the reservoirs for giardiasis in the NWT. Although epidemiological studies on human giardiasis have not to our knowledge been conducted in the NWT, 30% of Inuit on Baffin Island (Nunavut) were found to have laboratory-confirmed cases of the infection [5]. Furthermore, several outbreaks in Kodiak and Ketchikan (Alaska) were linked to the consumption of untreated surface water, thought to have been contaminated with cysts from the feces of beaver, hares and other carrier animals [5].

In our campylobacteriosis model, the statistically significant interaction between traditional foods and trapping suggests that in some instances, at the community level, activities that are thought to enhance exposure may have varying and complex effects on disease rates. We found that when the percentage of community participation in trapping was low, the risk of campylobacteriosis varied little with increasing percentages of traditional food consumption; however, when the percentage of community participation in trapping was higher, the protective effect against campylobacteriosis increased with greater consumption of traditional foods. One explanation for these results may be related to differential reporting between geographic regions. Higher consumption of traditional foods and higher rates of participation in trapping occur most often in more remote communities; however, it is also these communities that are often most disconnected from the reporting network of the surveillance system. Alternatively, it is possible that there is some form of traditional knowledge, genetic adaptation or acquired immunity (reflecting past exposure) that provides some protection to those frequently engaged in both activities. Maintaining a traditional lifestyle and diet are of great importance to the overall health and well-being of Aboriginal peoples [14]; therefore, targeted community-based collaborative research is required to more fully understand and interpret the complex relationships between these variables.

The combined challenges of storage, preparation, access and availability of traditional foods have caused communities to increase their consumption of retail foods as replacements for traditional foods; however, retail food prices are considerably higher in rural and remote communities compared to urban centers. A number of factors contribute to higher prices including freight charges, store management practices, and the reduced economies of scale for purchasing and retailing in small rural and remote communities [15,16]. Furthermore, the distance between producer and consumer corresponds to effectively shorter shelf life of perishable foods, raising the likelihood of spoilage and perhaps growth of pathogens (e.g., *Salmonella*) on fresh foods. Long distance transportation of fresh retail foods requires careful packing, handling, storage and distribution; thus, there are a number of sources from which microbial contamination can be contracted or be exacerbated as the pathogen loads multiply, thereby increasing the risk of illness [17]. In our model, a negative association was found between food prices in communities and risk of campylobacteriosis. Higher prices may affect food choices made by consumers, thereby changing their diet and diet-related health risks [15]. Households may reduce consumption of food items such as meat, dairy, poultry, fruits and vegetables which are more costly and opt for processed and packaged items, which could reduce exposure to pathogens more commonly found in perishable foods [15]. The association between higher food prices and decreased rates of campylobacteriosis may also be due to geographical reporting biases. Economically, traditional foods are still more affordable than retail foods, especially in remote areas [16]; thus, these communities tend to rely heavily on subsistence activities and may thereby have greater exposure to environmental contaminants and potential health risks [18-20].

Adequate, suitable and affordable housing is an essential component of health and well-being. A large body of scientific evidence demonstrates the association between housing quality and infectious diseases, chronic illnesses, and injuries [21-24]. In the North, the remote location combined with harsh climate creates higher costs and infrastructure needs [25]. Many northern, rural and remote communities face socioeconomic challenges such as relatively low income that can affect their access to adequate, suitable and affordable housing [26]. The 2001 Aboriginal Peoples Survey found that 33% of Aboriginal households are in core need, which is almost double the Canadian rate of 18% [27]. In our model, there was increased risk of infection with *Salmonella* for communities with higher proportions of households in core need up to 42% after which the rate started to decrease with increasing core need. Features of substandard housing which could facilitate exposure to and spread of *Salmonella* and other pathogens include poor water supply (quality and quantity) and sanitation (infrastructure) as well as inadequate food storage and preparation, and the intrusion of disease vectors (e.g., insects) [24,25]. The parabolic effect of households in core need (socioeconomic status) on salmonellosis may reflect complex interrelationships between food consumption patterns, safe food handling behaviors, contact with potential reservoir species of domestic and wild animals and other factors.

Person-to-person spread is not usually considered to be an important means of transmission of non-typhoidal salmonellosis but could be a factor in poor housing. High rates of respiratory illnesses in Aboriginal communities have also been attributed to inadequate and overcrowded housing. Tuberculosis rates in Aboriginal communities are 70 times the Canadian average [28]. Although the mode of transmission of tuberculosis and other respiratory infections differs from those derived from the environment, it does indicate that social determinants of health may play an important role in the communicability of these diseases.

Health care expenditure per capita is a leading indicator of the long-term sustainability of a health care system, with higher per capita costs indicating systems in difficulty [29]. The NWT has the second highest per capita expenditure in Canada as it operates four hospitals, 19 health centers, several nursing stations and a system-sponsored medical travel program for populations less than 50,000 spread across its 33 communities [30]. Between 2000 and 2006, the Canadian Institute for Health Information (CIHI) estimated that territorial government health care expenditures increased by 53% from $158 million to $242 million [31]. In our model, we observed that increases in health care expenditure were inversely related with *Giardia* rates. Increases in health care expenditure, in combination with existing health programs, may improve access, quality of care and services thereby reducing giardiasis and other disease burden in the territory; however, hypothesis-driven studies are necessary to better understand this association.

Our model also revealed a higher rate of reported giardiasis in communities where the primary health facility was a health center rather than a full-service hospital. Hospitals are located in Fort Smith, Hay River, Inuvik and Yellowknife whereas the other 29 communities are only serviced by health centers or remote nursing stations. Residents of these 29 communities are often unable to visit hospitals because of distance and/or reduced accessibility caused by geographic barriers or severe weather conditions [32]. Residents have reported that medical transfers to hospitals can be isolating and demoralizing experiences because of cultural and language barriers as well as separation from their families and communities [26]. As most cases of NGI are acute, self-limited and do not require hospitalization, they are more likely to be reported to health facilities in close proximity to the community. Giardiasis is largely a waterborne illness and therefore, it is also possible that the “communities with health center” variable is a surrogate for more rural and remote locations where the consumption of untreated surface water occurs more frequently than in urban centers where treated water supplies are readily available [6].

In summary, the linkage of the NWT Communicable Disease Registry and the NWT Community Survey provided a means of identifying potential community-level risk factors for NGI in the territory. Certain factors were associated with the increased likelihood of campylobacteriosis, giardiasis, and salmonellosis; however, these results should be interpreted with caution. There were several variables in the unconditional analysis that were associated with disease rates that were not included in the final model since they became insignificant in the presence of other factors. These variables should be considered potentially important as some associations may have been underestimated or undetected because of low statistical power. Given the interrelated nature of health determinants, it is also possible that risk factors in the final model were intervening variables (intermediate in the causal process between exposure and outcome) falsely decreasing the effect and significance of variables that were excluded from the model. We cannot rule out the role that geography might play in unbalanced disease diagnosis or reporting impacted by a complex mix of health care-seeking behaviors, access to health services, availability of diagnostic tests, and reporting practices by health professionals and laboratories. The rates of infections reported in this study are likely underestimates of the true incidence of diseases and therefore, should be interpreted as reporting rates rather than as incidence rates [33]. Currently, the disease data that are available are not sufficient nor appropriate to address underdiagnosis or underreporting in the NWT; however, geographically identified case–control studies, sentinel surveillance, community-based programs and knowledge, attitude and practice surveys, could be used to identify and quantify these biases (if any exist) in the future. There were also many factors that could not be evaluated because of the limited suite of epidemiological information that accompanies the notification. Furthermore, it was not possible to take person-level risk factors into account. Analytical studies, particularly cohort and case–control studies would be required to assess the role of person-level factors, such as age and sex, as well as person and household-level exposure to various foods, water and other environmental factors. Additionally, since this is an ecological study, drawing inferences at the person level is inappropriate and may lead to biased interpretation.

## Conclusions

The results of this study support the view that community-based programs that address safe water and food supplies as well as adequately designed and appropriately maintained housing can play a key role in disease prevention and control. Our findings also point to several hypotheses that could be addressed in the future using a prospective, multilevel analytic approach that combines person and contextual community-level factors. Although expensive and logistically challenging to conduct in remote communities with sparse populations, such research would provide more definitive insight into individual health outcomes that would be useful in crafting policies aimed at reducing health inequalities and thus costs, in the long term. Further study of subsistence activities, food preparation and consumption patterns as well as medical care-seeking behaviors across communities can elucidate whether observed distribution of disease is due to underreporting or underlying risk or protective factors. These findings can assist in targeting further investigations that can ultimately lead to strategic interventions to reduce the incidence of NGI in groups at high risk.

## Abbreviations

NGI: Notifiable gastrointestinal illness; NWT: Northwest Territories; NWT CDR: Northwest Territories Communicable Disease Registry; SAS: Statistical Analysis System; CIHI: Canadian Institute for Health Information.

## Competing interests

The authors declare that they have no competing interests.

## Authors’ contributions

AP-A contributed to the manuscript through study design and planning, data collection, analysis and interpretation of results, drafting of manuscript and response to editorial comments and preparation of final manuscript for submission. JW, VLE, CF, RRS and SAM contributed to the manuscript through study design and planning, consultation on study progress, troubleshooting, data analysis and interpretation of results, reviewing and commenting on manuscript drafts. MS contributed to the manuscript through data collection, interpretation of results and reviewing and commenting on manuscript drafts. All authors read and approved the final manuscript.

## Pre-publication history

The pre-publication history for this paper can be accessed here:

http://www.biomedcentral.com/1471-2458/13/63/prepub
